# Perturbed proteostasis in autism spectrum disorders

**DOI:** 10.1111/jnc.13723

**Published:** 2016-08-04

**Authors:** Susana R. Louros, Emily K. Osterweil

**Affiliations:** ^1^ Centre for Integrative Physiology/Patrick Wild CentreUniversity of EdinburghHugh Robson BuildingGeorge SquareEdinburghEH8 9XDUK

**Keywords:** ASD/ID, autism, proteasome, synaptic plasticity, translation, ubiquitin

## Abstract

Dynamic changes in synaptic strength rely on *de novo* protein synthesis and protein degradation by the ubiquitin proteasome system (UPS). Disruption of either of these cellular processes will result in significant impairments in synaptic plasticity and memory formation. Mutations in several genes encoding regulators of mRNA translation and members of the UPS have been associated with an increased risk for the development of autism spectrum disorders. It is possible that these mutations result in a similar imbalance in protein homeostasis (proteostasis) at the synapse. This review will summarize recent work investigating the role of the UPS in synaptic plasticity at glutamatergic synapses, and propose that dysfunctional proteostasis is a common consequence of several genetic mutations linked to autism spectrum disorders.

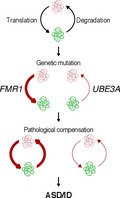

Dynamic changes in synaptic strength rely on *de novo* protein synthesis and protein degradation by the ubiquitin proteasome system (UPS). Disruption of either of these cellular processes will result in significant impairments in synaptic plasticity and memory formation. Mutations in several genes encoding regulators of mRNA translation (i.e. *FMR1*) and protein degradation (i.e. *UBE3A*) have been associated with an increased risk for autism spectrum disorders and intellectual disability (ASD/ID). These mutations similarly disrupt protein homeostasis (proteostasis). Compensatory changes that reset the rate of proteostasis may contribute to the neurological symptoms of ASD/ID. This review summarizes recent work investigating the role of the UPS in synaptic plasticity at glutamatergic synapses, and proposes that dysfunctional proteostasis is a common consequence of several genetic mutations linked to ASD.

This article is part of a mini review series: “Synaptic Function and Dysfunction in Brain Diseases”.

**Abbreviations used**
αCaMKII: αCa^2+^/calmodulin‐dependent kinase II
AMPAR: α‐amino‐3‐hydroxy‐5‐methyl‐4‐isoxazolepropionic acid receptor
Arc/Arg3.1: activity‐regulated cytoskeleton‐associated protein
AS: Angelman syndrome
ASD: autism spectrum disorders
BDNF: brain‐derived neurotropic factor
CA1: *Cornu Ammonis* 1
Cdh1‐APC: anaphase‐promoting complex ubiquitin ligase
CNVs: copy number variants
CPEB3: cytoplasmic polyadenylation element binding protein 3
DUB: de‐ubiquitinase
FMRP: fragile X mental retardation protein
FXS: fragile X syndrome
GFP: green fluorescent protein
GKAP: guanylate kinase‐ associated protein
IA: inhibitory avoidance
ID: intellectual disability
LTD: long‐term depression
LTP: long‐term potentiation
MAPK: ras‐mitogen activated protein kinase
mGluR1/5: group I metabotropic glutamate receptors
Mib2: mind bomb 2 ubiquitin ligase
mTOR: mammalian target of rapamycin
PSD95: post‐synaptic density protein of 95 KDa
Siah1a: seven *in absentia* homolog 1A
SPAR: rap GTP activating protein
TSC: tuberous sclerosis complex
UPS: ubiquitin proteasome system

## Protein synthesis and degradation in ASD/ID

Functioning neural circuits require synaptic connections capable of strengthening or weakening in response to activity. This plasticity, measured electrophysiologically as the long‐term potentiation or depression of synaptic strength (LTP/D), is particularly important for experience‐dependent memory formation. It is well established that *de novo* protein synthesis plays a fundamental role in supporting LTP/D and that it is required for the creation of new memories. Given this important role in synaptic plasticity, it is perhaps not surprising that mutations in several genes that encode regulators of protein synthesis have been identified as risk factors for the development of autism spectrum disorders with accompanying intellectual disability (ASD/ID) (Table**** 1) (Kelleher and Bear 2008; Bhakar *et al*. 2012). These include mutations in the *FMR1* and *TSC1* or *TSC2* genes, which respectively give rise to the neurodevelopmental disorders fragile X syndrome and tuberous sclerosis complex (TSC), as well as many regulators of the Ras‐MAPK and mammalian target of rapamycin (mTOR) translation control pathways (Kelleher and Bear 2008; Krab *et al*. 2008). Studies in animal models of these disorders reveal that protein synthesis downstream of group I metabotropic glutamate receptors (mGluR1/5) is commonly disrupted, leading to dysfunctional long‐term depression (LTD) (Dolen *et al*. 2007; Krab *et al*. 2008; Osterweil *et al*. 2010; Auerbach *et al*. 2011; Bateup *et al*. 2011; Barnes *et al*. 2015). Subsequent studies in multiple other mouse models of ASD/ID reveal a similar dysregulation of protein synthesis and LTD (Table**** 1). Importantly, normalizing mRNA translation corrects aberrant synaptic plasticity and several other pathological phenotypes in many of these mutant models.

**Table 1:** ASD/ID mutations in genes encoding regulators of mRNA translation

Gene	Disorder	Function	Phenotypes	References
FMR1	Fragile X syndrome (ID, ASD)	Translation repressor	Enhanced mGluR‐LTD Impaired LTP Impaired learning and memory	Reviewed in Bhakar *et al*. (2012) and Darnell and Klann (2013)
CYFIP1	ASD	Translation repressor	Enhanced mGluR‐LTD Enhanced extinction of inhibitory avoidance	Nishimura *et al*. (2007), Bozdagi *et al*. (2012) and Wang *et al*. (2015)
SYNGAP1	ID, ASD	Ras‐MAPK negative regulator	Enhanced mGluR‐LTD Impaired LTP Learning and memory deficits	Komiyama *et al*. 2002, Barnes *et al*. 2015 and Jeyabalan and Clement (2016)
NF1	Neurofibromatosis type 1 (ID)	Ras‐MAPK negative regulator	Impaired LTP Abnormal spatial learning	Silva *et al*. (1997), Costa *et al*. (2002) and Sanders *et al*. (2011)
TSC1/2	Tuberous sclerosis complex (ID, ASD)	Rheb‐mTOR negative regulator	Impaired mGluR‐LTD Abnormal LTP Learning and memory deficits	Ehninger *et al*. (2008) and Auerbach *et al*. (2011)
PTEN	Cowden syndrome (ID, ASD)	PI3K‐mTOR negative regulator	Impaired LTP and LTD Impaired spatial memory	Butler *et al*. (2005), Kwon *et al*. (2006) and Sperow *et al*. (2012)
RPL10	ID, ASD	Ribosomal protein	ND	Klauck *et al*. (2006), Brooks *et al*. (2014) and Thevenon *et al*. (2015)
RPS6KA2	ASD	Ribosomal p90 S6 kinase (MAPK pathway)	ND	Marshall *et al*. (2008)
RPS6KA3	ID, ASD	Ribosomal p90 S6 kinase (MAPK pathway)	Impaired spatial learning	Zeniou *et al*. (2002), Zeniou‐Meyer *et al*. (2010), O'Roak *et al*. (2012) and Matsumoto *et al*. (2013)
EIF4E	ASD	Initiation factor	Enhanced mGluR‐LTD Impaired social behavior Repetitive behaviors	Neves‐Pereira *et al*. (2009), Kelleher *et al*. (2012), Gkogkas *et al*. (2013) and Santini *et al*. (2013)
EEF1A2	ASD/ID	Elongation factor	ND	de Ligt *et al*. (2012) and Nakajima *et al*. (2015)
RBMS3	ASD	RNA binding protein	ND	O'Roak *et al*. (2011)
HRAS	Costello syndrome (ASD)	Ras GTPase	Enhanced LTP Enhanced spatial learning Enhanced fear conditioning	Herault *et al*. (1993, 1995), Comings *et al*. (1996), Manabe *et al*. (2000), Kushner *et al*. (2005), Kelleher *et al*. (2012) and Alfieri *et al*. (2015)
BRAF	Costello syndrome/Noonan syndrome (ID, ASD)	MAPK activator	Impaired LTP Impaired spatial learning Impaired contextual discrimination	Chen *et al*. (2006) and Alfieri *et al*. (2014)
PTPN11	Noonan syndrome (ID)	Ras pathway regulator	Impaired LTP Impaired spatial learning	Tartaglia *et al*. (2001), Lee *et al*. (2014), Deciphering Developmental Disorders (2015) and Krumm *et al*. (2015)
SOS1	Noonan syndrome (ID)	Ras pathway regulator	ND	Roberts *et al*. (2007) and Tartaglia *et al*. (2007)

Several genetic mutations that confer risk for developing ASD or ID are found in genes related to protein synthesis. These include regulators of the Ras‐MAPK and mTOR signaling pathways that control mRNA translation at synapses. Synaptic plasticity and learning phenotypes are seen in mouse models of many of these disorders (ND = not determined).

**Figure 1:**
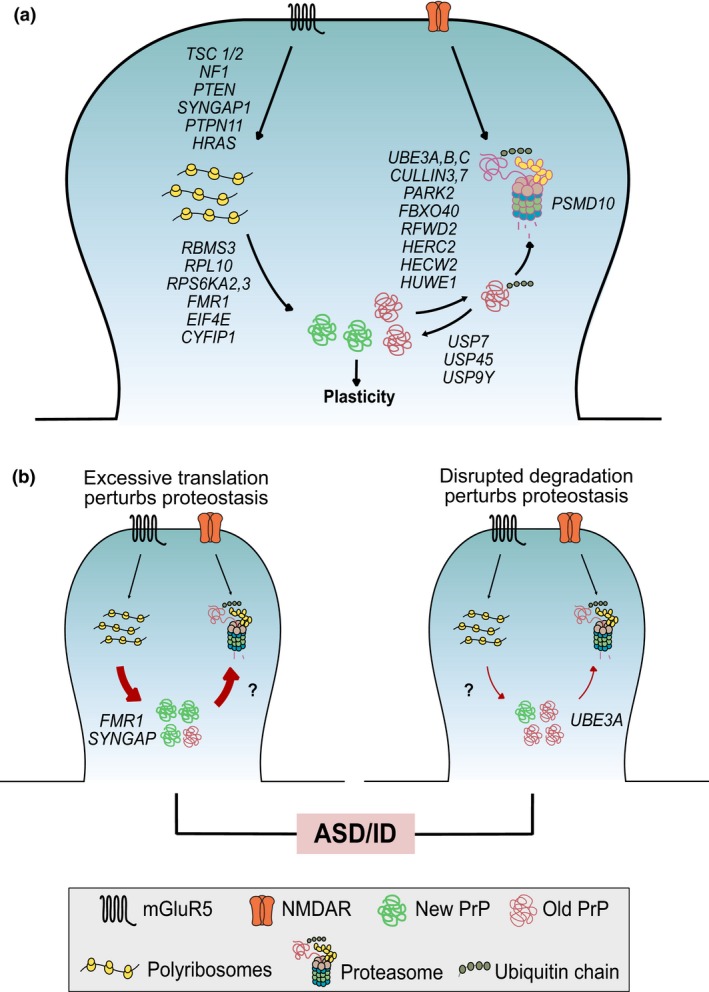
Dysregulation of protein synthesis or degradation results in unbalanced proteostasis. (a) Mutations in several genes that regulate mRNA translation and ubiquitin proteasome system function have been implicated in ASD/ID (see Tables 1, 2). This includes regulators of translation control signaling pathways (*TSC1/2, NF1, PTEN, SYNGAP1, PTPN11, HRAS*), protein synthesis regulators (*FMR1, CYFIP1, EIF4E, RBMS3, RPL10, RPSS6KA2,3*), E3 ubiquitin ligases (*UBE3A,B,C, CULLIN3,7, PARK2, FBXO40, RFWD2, HERC2, HECW2, HUWE1*), de‐ubiquitinases (*USP7, USP45, USP9Y*), and the proteasome protein *PSMD10*. The proteins encoded by these genes collectively contribute to the proteostasis involved in synaptic plasticity. (b) The pathogenic excess in synaptic protein synthesis observed in animal models of ASD/ID (i.e. *FMR1*,*SYNGAP1*, and *CYFIP1*) may lead to a homeostatic increase in UPS function. Similarly, mutations in E3 ligases, such as Ube3A, that decrease UPS function may result in a compensatory decrease in protein synthesis. In both cases, the imbalance in proteostasis would lead to a change in the composition of new versus old plasticity related proteins (PrPs) in the synaptic proteome without necessarily affecting overall protein levels.

Interestingly, the changes in protein synthesis observed in many mouse models of ASD/ID do not appear to be accompanied by significant changes in protein expression. One explanation is that there is a compensatory change in the rate of protein degradation in order to prevent large shifts in the abundance of the synaptic proteome. If so, it may be that this in itself contributes to the neurological phenotypes seen in these mutant models. Indeed, the coordination between protein synthesis and breakdown of proteins by the ubiquitin proteasome system (UPS) is thought to play an important role in the regulation of synaptic function and plasticity (Hanus and Schuman 2013). Although the role of the UPS in neurodevelopmental disorders has received relatively little attention, one of the most commonly mutated genes linked to ASD/ID encodes the ubiquitin E3 ligase Ube3a (Kishino *et al*. 1997). Moreover, mutations in over a dozen other UPS genes have been identified as risk factors for ASD/ID (Table**** 2). An intriguing possibility is that imbalance in the combined process of protein synthesis and breakdown (proteostasis) could be a common contributor to the development of ASD/ID (Fig. 1).

**Table 2:** ASD/ID risk factors in ubiquitin proteasome system (UPS) genes

Gene	Disorder	Function	Phenotypes	References
UBE3A	Angelman syndrome (ID, ASD), ASD	E3 ubiquitin ligase	Enhanced mGluR‐LTD Impaired LTP Deficits in contextual learning	Jiang *et al*. (1998) and Pignatelli *et al*. (2014)
UBE3B	ASD	E3 ubiquitin ligase	ND	Basel‐Vanagaite *et al*. (2012), Chahrour *et al*. (2012) and Flex *et al*. (2013)
UBE3C	ASD	E3 ubiquitin ligase	ND	O'Roak *et al*. (2012)
UBR7	ID	E3 ubiquitin ligase	ND	Najmabadi *et al*. (2011)
PARK2	ASD	E3 ubiquitin ligase	ND	Glessner *et al*. (2009)
FBXO40	ASD	E3 ubiquitin ligase	ND	Glessner *et al*. (2009)
RFWD2	ASD	E3 ubiquitin ligase	ND	Glessner *et al*. (2009)
Cullin 3	ASD	E3 ubiquitin ligase	ND	O'Roak *et al*. (2012) and Codina‐Sola *et al*. (2015)
Cullin 7	ASD	E3 ubiquitin ligase	ND	Krumm *et al*. (2015)
HECW2	ASD	E3 ubiquitin ligase	ND	Krumm *et al*. (2015)
HERC2	ASD	E3 ubiquitin ligase	ND	Puffenberger *et al*. (2012) and Harlalka *et al*. (2013)
HUWE1	ID, ASD	E3 ubiquitin ligase	ND	Froyen *et al*. (2008, 2012), Nava *et al*. (2012) and Vandewalle *et al*. (2013)
UBL7	ASD	Ubiquitin binding protein	ND	Salyakina *et al*. (2011)
PSMD10	ASD	Proteasome protein	ND	Piton *et al*. (2011)
USP9Y	ASD	De‐ubiquitinase	ND	ND
USP45	ASD	De‐ubiquitinase	ND	ND
USP7	ASD	De‐ubiquitinase	ND	ND

Mutations in several UPS genes have been identified as risk factors for ASD or ID. These include multiple genes encoding ubiquitin E3 ligases and deubiquitinases that regulate protein degradation. With the exception of mutations in *UBE3A*, the functional consequences of these gene mutations have not been determined (ND).

In this review, we will summarize work linking ubiquitination and proteasome activity to changes in synapse function. Our emphasis will be on studies investigating the role of the UPS in the plasticity of excitatory synapses that contribute to learning and memory. The links between UPS dysfunction, protein synthesis, and the development of ASD/ID will be discussed.

## UPS regulation by synaptic activity

The process of protein degradation is essential for the function of all eukaryotic cells, including neurons. Pathologically misfolded proteins must be removed, short‐lived proteins must be quickly degraded in response to activity, and more stable constituents must be turned over to maintain the infrastructure of the cell. The majority of cytosolic and nuclear proteins are degraded by the UPS, which is comprised of the 26S proteasome and the ubiquitin ligases that tag proteins for degradation (for extensive review see Weissman 2001; Schmidt and Finley 2014). The proteasome consists of multi‐subunit 19S regulatory particles, and a 20S catalytic core that hydrolyzes ATP in order to break down target proteins (Weissman 2001). For recognition by the proteasome, proteins destined for degradation must be tagged with a polyubiquitin chain. The covalent attachment of ubiquitin to the target protein involves three different enzymes: the E1 ligase that activates monoubiquitin, the E2 ligase that conjugates additional ubiquitin monomers to form a chain, and finally the E3 ligase that selectively conjugates the polyubiquitin chain to its target protein (Schmidt and Finley 2014; Suryadinata *et al*. 2014). It is the affinity of E3 ligases for select protein targets that determines the specificity of the UPS for only those proteins that require degradation. Interestingly, the addition of a single ubiquitin to a target protein, rather than a polyubiquitin chain, may serve as a tag for intracellular trafficking rather than degradation. Generally, proteins bound to lysine 48 (K48) chains are directly targeted for proteasomal degradation while a lysine 63 (K63) chain or a single ubiquitin molecule may result in significant effects in subcellular localization or activity of proteins (Suryadinata *et al*. 2014).

In neurons, the UPS has been implicated in several fundamental processes including morphogenesis, dendritic spine structure, synaptic activity, and the regulation of synaptic strength (for excellent reviews of these topics see Bingol and Sheng 2011; Tai *et al*. 2010; Hegde 2010; Hamilton and Zito 2013). Several lines of evidence show that the proteasome is regulated by neuronal activity through alterations in four factors: subunit composition, proteolytic activity, location within the cell, and interaction with other proteins. Pioneering studies in cultured neurons showed that manipulation of neuronal activity resulted in a dramatic change in the ubiquitination and degradation of the post‐synaptic proteome (Ehlers 2003). Subsequent work revealed that proteasomes can be translocated from dendritic shafts into post‐synaptic dendritic spines within minutes upon KCl‐induced depolarization, leading to overall increased local proteolysis (Bingol and Schuman 2006). More recent studies expressing a proteasome substrate GFP^u^ in cultured hippocampal neurons demonstrated that proteasomal breakdown is directly related to network activity. Blockade of action potential firing with the sodium channel blocker tetrodotoxin decreased the degradation rate of GFP^u^, whereas increasing neuronal activity with the GABA‐A receptor antagonist bicuculline lead to more degradation (Djakovic *et al*. 2009).

The majority of studies focused on UPS activity in synaptic function have examined excitatory neurotransmission through the activation of ion channel linked NMDA‐type glutamate receptors (NMDARs) and α‐amino‐3‐hydroxy‐5‐methyl‐4‐isoxazolepropionic acid receptors (AMPARs) at the post‐synaptic membrane. Experiments in cultured neurons show that agonist‐induced activation of NMDARs leads to disassembly of the 26S proteasome, resulting in decreased proteolytic activity and a dissociation of E3 ligases from the proteasome (Tai *et al*. 2010). In addition, it may be that the NMDAR acts to stabilize proteasomes at synapses in order to facilitate the trafficking of AMPARs. Proteomic examination of hippocampal neurons from mice lacking the NMDAR subunit GluN2B reveals a decrease in the abundance of several proteasome subunits in purified post‐synaptic density fractions (Ferreira *et al*. 2015).

NMDARs also regulate UPS function through the activation of the abundant post‐synaptic protein α‐Ca^2+^/calmodulin‐dependent kinase II (αCaMKII). Evidence from cultured neurons shows that Ca^2+^‐dependent activation of αCaMKII through either NMDARs or L‐type voltage‐gated Ca^2+^ channel results in an increased association with proteasomes, and the recruitment of proteasomes into synapses (Djakovic *et al*. 2009). The stimulation of proteasome activity by αCaMKII involves phosphorylation of the regulatory subunit Rpt6, which increases the catalytic activity of the 20S core (Bingol *et al*. 2010). Evidence showing that αCaMKII itself can be ubiquitinated suggests that its effects on the proteasome could be autoregulatory (Na *et al*. 2012), however, the specific E3 ligases regulating this process remain unknown.

## UPS regulation of the post‐synaptic proteome

Several studies have shown that manipulation of the UPS has a significant impact on both the structure and function of the post‐synaptic compartment. Recent studies using 2‐photon live imaging in organotypic hippocampal slice culture showed that pharmacological inhibition of the UPS dramatically reduces the rate of dendritic spine outgrowth (Hamilton *et al*. 2012). This regulation of spine outgrowth by the UPS was seen to rely on the interaction between GluN2B and αCaMKII. Although the UPS substrates involved in dynamic spine morphogenesis have not been extensively characterized, the ubiquitination of the actin regulatory protein spine‐associated Rap GTP activating protein (SPAR) has been implicated in dendritic spine shrinkage and synapse weakening (Pak *et al*. 2001).

The UPS regulates numerous proteins that have a direct impact on synaptic transmission. This includes major post‐synaptic scaffolding proteins such as post‐synaptic density protein of 95 KDa (PSD‐95), guanylate kinase‐ associated protein (GKAP) and Shank, as well as major plasticity‐related proteins (PrPs) such as Arc/Arg3.1 and αCaMKII (Ehlers 2003). Additionally, NMDARs and AMPARs themselves are major UPS substrates, as are a number of other neurotransmitter receptors including mGluR1/5, inhibitory GABARs, kainate receptors, glycine receptors, nicotinic acetylcholine receptors, and dopamine receptors (Lin and Man 2013). Systematic analysis of the ubiquitome in the adult rat brain using a new monoclonal antibody to purify ubiquitinated peptides followed by mass spectrometry revealed a wide range of ubiquitination events on 45 key components of the pre‐synaptic region and of the post‐synaptic density including PSD‐95, CaMKII, and receptors for AMPA, NMDA, GABA, serotonin, and acetylcholine. Interestingly, several UPS proteins were also found to be ubiquitinated, including E1, E2, E3 ligases, 10 DUBs, and several proteasome subunits (Na *et al*. 2012).

Degradation of the NMDAR is mediated by ubiquitination of the obligatory GluN1 subunit by the E3 ligase Fbxo2, which directs receptors to cytosolic proteasomes (Kato *et al*. 2005). The GluN2B subunit of the NMDAR is also ubiquitinated by the E3 ligase mind bomb 2 (Mib2) (Jurd *et al*. 2008). Both mGluR1a and mGluR5 can be ubiquitinated by the E3 ligase seven in absentia homolog 1A leading to proteasomal degradation (Moriyoshi *et al*. 2004). The ubiquitination of mGluR1a may be regulated by synaptic plasticity, as it has been shown to require the association with the Homer3 scaffolding protein (Rezvani *et al*. 2012).

One of the most well‐described post‐synaptic targets of the UPS is the AMPAR. A recent study showed that all four AMPAR subunits (GluA1‐4) are rapidly ubiquitinated upon brief application of AMPA or bicuculline in cultured neurons (Widagdo *et al*. 2015). The increase in neuronal activity leads to ubiquitination of GluA1 by the E3 ligase Nedd4‐1, which internalizes AMPARs and directs them to endosomes and lysosomes for degradation (Schwarz *et al*. 2010). This process can be counteracted by the deubiquitinase (DUB) USP8 in response to NMDAR activation, which promotes AMPAR reinsertion into the post‐synaptic membrane (Scudder *et al*. 2014). This study also reported direct modulation of E3 ligases and DUBs by synaptic activity. Upon AMPAR stimulation, Nedd4‐1 is rapidly redistributed to dendritic spines while NMDAR stimulation selectively activates USP8. Therefore, Nedd4‐1 and USP8 are regulated at synapses to control synaptic strength in an opposite fashion, regulating AMPAR ubiquitination and function. Moreover, bicuculline‐induced downscaling of AMPARs and synaptic strength is accompanied by an increase in Nedd4‐1 and a decrease in USP8 protein levels, respectively, showing that E3 ligases and DUBs can be modulated during Hebbian and homeostatic plasticity (Scudder *et al*. 2014). Recently*, in vivo* and *in vitro* studies demonstrated that the DUB USP46 also targets GluA1, regulating AMPAR surface expression, endocytosis, and the strength of synaptic transmission (Huo *et al*. 2015). During homeostatic plasticity, GluA1 is also targeted by the E3 ligase Cdh1‐APC and degraded by the proteasome, a process that involves signaling through ephrin receptor EphA4 (Fu *et al*. 2011). Altogether, these studies indicate that ubiquitination is an important regulatory signal for controlling AMPAR function. This may explain the observed importance of the UPS for maintaining LTP and LTD, both of which occur through changes in AMPAR trafficking.

## The role of the UPS in synaptic plasticity

Early experiments performed in *Aplysia* revealed a critical role for the UPS in the long‐term facilitation of synaptic strength at sensory‐motor synapses (Hegde *et al*. 1997). This observation was supported by subsequent experiments in rat hippocampal *Cornu Ammonis* 1 (CA1) showing that the proteasome inhibitor MG132 blocks both the early protein synthesis‐independent phase of LTP (E‐LTP), and the late‐phase LTP (L‐LTP) that requires protein synthesis (Karpova *et al*. 2006). Later experiments revealed that application of the specific proteasome inhibitor lactacystin enhanced E‐LTP but blocked L‐LTP at hippocampal CA1 synapses (Fonseca *et al*. 2006; Dong *et al*. 2008). The specific effect of proteasome inhibitors on L‐LTP but not E‐LTP suggested that the requirement for protein degradation was related to the requirement for new protein synthesis. Further studies proved this correct, showing that the augmentation of LTP by proteasome inhibitors is blocked by the presence of the protein synthesis inhibitor anisomycin. This indicated that proteasome inhibition increases the induction of LTP by stabilizing locally translated proteins in dendrites (Dong *et al*. 2008). Supporting this interpretation, a recent study linked the enhancement of E‐LTP by proteasome inhibition to increases in the levels of the translation initiation factors eIF4E and eF1A (Dong *et al*. 2014). This study further suggested that proteasome inhibition might impair the consolidation of L‐LTP because of an accumulation of Paip2 and 4E‐BP2, two translational repressors (Dong *et al*. 2014).

Another way in which the UPS may modulate LTP is in the regulation of brain‐derived neurotropic factor (BDNF). Recent work showed that application of BDNF induced a rapid and transient decrease in proteasome activity in hippocampal synaptoneurosome fractions, and that the proteasome activator IU1 blocked the enhancement of E‐LTP by BDNF (Santos *et al*. 2015). Similar to previous studies, the authors show that proteasome inhibitors block the expression of L‐LTP and the effect of BDNF upon LTP consolidation (Santos *et al*. 2015). These results support earlier findings, and underscore the conclusion that the combination of both the degradation and synthesis of proteins is required to support the long‐term strengthening of synapses.

The induction of LTD at hippocampal CA1 synapses can be induced by either the weak stimulation of NMDARs or through stimulation of mGluR1/5, both of which elicit changes in synaptic efficacy through AMPAR endocytosis (Dudek and Bear 1992; Huber *et al*. 2001). Although they can occur at the same set of synapses, a major distinction is that mGluR‐LTD requires new protein synthesis, whereas NMDAR‐LTD does not (Huber *et al*. 2000). Interestingly, the role of the UPS may also differentiate these forms of LTD. Multiple studies have shown that proteasome inhibitors reduce the AMPAR endocytosis and LTD downstream of NMDAR activation (Colledge *et al*. 2003; Patrick *et al*. 2003; Bingol and Schuman 2004; Citri *et al*. 2009). The role of the UPS in mGluR‐LTD, however, is not as clear. Initial studies showed that incubation of hippocampal slices with the proteasome inhibitors MG132 or lactacystin resulted in impairment of mGluR‐LTD (Hou *et al*. 2006). The authors proposed that the UPS sensitivity of mGluR‐LTD resulted from breakdown of the translation repressor fragile X mental retardation protein, the protein lost in fragile X syndrome (Hou *et al*. 2006). Recent work identifies Cdh1‐APC as the E3 ligase responsible for fragile X mental retardation protein degradation, and shows that mice lacking this ligase exhibit impaired mGluR‐LTD (Huang *et al*. 2015). Along the same lines, another recent study showed that mGluR‐LTD requires the rapid degradation of Arc/Arg3.1, a process that is counterbalanced by the RNA binding protein Sam68 (Klein *et al*. 2015). Together, these results support the idea that the UPS is required for the induction of mGluR‐LTD.

In contrast to these results, other studies find that the UPS may be inhibitory for mGluR‐LTD. A study directly comparing both forms of LTD showed that the proteasome inhibitors MG132 and lactacystin inhibit NMDAR‐LTD but enhance mGluR‐LTD. Additionally, application of UBEI‐41/PYR‐41, a cell‐permeable compound that irreversibly inhibits the E1 activating enzyme, was shown to enhance both the AMPAR internalization and mGluR‐LTD (Citri *et al*. 2009). Other work shows that application of proteasome inhibitors enhances the transition from early‐to late‐phase LTD (Li *et al*. 2015). The seemingly dual nature of the UPS in LTD may result from the regulation of different target proteins: those that are ubiquitinated to facilitate the induction of LTD and those that are broken down to limit the extent of LTD. The identification of these target proteins may clarify the opposing results regarding the function of the UPS during long‐term plasticity.

## Requirement of the UPS for learning and memory

The key role of proteasomal degradation in the expression of long‐term synaptic plasticity has led to investigation of the UPS in learning and memory. A variety of different behavioral paradigms have been used to study the impact of synaptic plasticity on learning and memory formation. Using these paradigms, it has been shown that new mRNA translation facilitates memory formation by stabilizing molecular and synaptic changes during both consolidation (after learning) and reconsolidation (after memory reactivation) (reviewed in Jarome and Helmstetter 2014). In order to determine the role of the UPS in these aspects of memory formation, initial studies tested the effects of lactacystin infused into the hippocampus after training on an inhibitory avoidance learning task. The results showed that lactacystin infusion resulted in a full retrograde amnesia, similar to what is seen with protein synthesis inhibitors. Concomitantly, inhibitory avoidance training resulted in an increase in protein ubiquitination and UPS activity in the hippocampus. These findings were the first to indicate that the UPS is crucial for the establishment of long‐term memory in rats (Lopez‐Salon *et al*. 2001). Since then, it has been demonstrated that the inhibition of the proteasome in multiple brain regions results in impairments in memory consolidation (Jarome and Helmstetter 2014).

The reconsolidation of memory is also sensitive to proteasome inhibition. Studies in the hippocampus showed that infusion of lactacystin blocked the extinction of fear conditioning and prevented the memory‐impairing effect of the protein synthesis inhibitor anisomycin when given after retrieval, but did not affect memory formation when administered after training. Based on this, the authors proposed that the UPS is required for the destabilization of pre‐existing memories, allowing for modification by reconsolidation or extinction (Lee *et al*. 2008). However, this conflicts with more recent work showing that both consolidation and reconsolidation depend on protein synthesis and also on protein degradation by UPS (Figueiredo *et al*. 2015).

Nevertheless, there is a clear relationship between the requirement for new protein synthesis and UPS function in the acquisition of memory. Indeed, the time at which memory retention is sensitive to proteasome inhibitors is the same 3–4 h post‐acquisition time window that is sensitive to protein synthesis inhibitors (Bourtchouladze *et al*. 1998; Figueiredo *et al*. 2015). One possibility is that the degradation of translation inhibitors is needed to promote protein synthesis‐dependent plasticity (Bingol and Sheng 2011). It is also possible that the non‐proteolytic function of the UPS is required. Supporting this notion, the monoubiquitination of the translation regulator cytoplasmic polyadenylation element binding protein 3 was shown to be critical for the consolidation of hippocampus‐dependent memories (Pavlopoulos *et al*. 2011). Further work is needed to understand the precise mechanisms by which the UPS contributes to the formation of new memories, and the potential regulation of protein synthesis.

## UPS mutations in ASD/ID

The impact of proteasome dysfunction on human cognition has been an active field of research with respect to neurodegenerative disorders such as Alzheimer's, Parkinson's, and Huntington's disease. In these disorders, it is widely accepted that proteasomal dysfunction is, at least in part, responsible for the formation of protein inclusions in specific neuronal subtypes that ultimately will cause neurodegeneration (Dantuma and Bott 2014). However, very little is known about the role of the UPS in neurodevelopmental disorders.

One notable exception to this is Angelman syndrome (AS), a neurodevelopmental disorder caused by disruption of the E3 ligase Ube3a, which is characterized by ID, developmental delay, seizures, motor disruptions, and an unusually positive demeanor (LaSalle *et al*. 2015). While the majority of cases are caused by the specific loss of the maternal *UBE3A* allele, several studies have shown that mutations affecting the catalytic domain of Ube3a can also result in AS symptomology (Kishino *et al*. 1997; Matsuura *et al*. 1997; Cooper *et al*. 2004). Missense mutations targeting an inhibitory phosphorylation site on Ube3a have also been identified as risk factors for developing ASD/ID (Yi *et al*. 2015). Interestingly, the duplication or triplication of the chromosomal region 15q11‐q13 in which the *UBE3A* gene resides is a major cytogeneic risk factor for ASD (LaSalle *et al*. 2015). While it is not clear that this is due directly to the increased level of Ube3a, transgenic mice engineered to express multiple copies of the *Ube3a* gene exhibit impaired social behavior and communication, and increased repetitive behaviors (Smith *et al*. 2011). Together, these studies strongly suggest that misregulation of Ube3a function is a causative factor in the development of ASD/ID.

Studies of the *Ube3a* ^m‐/p+^ mouse model reveal several neuropathological changes reminiscent of AS, including deficits in synaptic plasticity in several brain regions and significant impairments in learning and memory (Mabb *et al*. 2011). Given the many neurological phenotypes associated with changes in Ube3a expression, one major task in the field has been to identify brain‐derived targets as disease‐relevant substrates (LaSalle *et al*. 2015). The most studied Ube3a substrate is Arc/Arg3.1, a cytoskeleton‐associated protein known to regulate trafficking of AMPARs to the membrane (Greer *et al*. 2010). Interesting new work from Kuhnle and colleagues proposes an alternate mechanism by which Ube3a regulates Arc expression in immortalized cell lines. In this study, the authors suggest that Ube3a negatively regulates Arc expression at the transcription level, rather than at the posttranslational level. In fact, the use of the dihydrofolate reductase‐ubiquitin fusion protein system confirmed that over‐expression of E6AP did not significantly affect the ubiquitination status or the levels or Arc (Kuhnle *et al*. 2013). Although these results have yet to be verified in neuronal cells, this is an important study because it shows a new level of regulation of Arc expression by Ube3a. However, this would seem to conflict with a recent study which demonstrated that reduction of Arc levels in the *Ube3a* ^m−/p+^ mouse model ameliorated some phenotypes presented by the model of Angelman Syndrome (Mandel‐Brehm *et al*. 2015). More research into the regulation of Arc is certain to reveal the relationship between these results. Indeed, Arc degradation by the UPS is also regulated by Triad3A (Mabb *et al*. 2014) showing a complex mechanism of regulation of Arc protein levels.

Another interesting target of Ube3a is the mTOR suppressor protein Tsc2 (Zheng *et al*. 2008). Recent work suggests that breakdown of Tsc2 by Ube3a may contribute to pathology in the *Ube3a* ^m−/p+^ mouse of AS, as treatment with the mTOR inhibitor rapamycin rescued motor deficits and abnormal dendritic branching (Sun *et al*. 2015). Despite these results, it is important to consider that the Ube3a substrates described so far contribute to only a subset of phenotypes associated with AS. It may be that the key Ube3a substrates have not yet been identified or, more likely, that the disruption of Ube3A leads to a multiplicative effect involving multiple downstream targets. Indeed, recent *in vitro* studies reveal that Ube3a promotes the ubiquitination of the 26S proteasome itself, suggesting that it can have a significant impact on overall UPS function (Jacobson *et al*. 2014).

In addition to *UBE3A*, mutations in over a dozen other UPS genes, mainly E3 ligases, have been identified as ASD/ID risk factors (Table**** 2). Large‐scale studies have identified copy number variations that result in the deletion of the E3 ligase gene *PARK2*, and duplication in the E3 ligase genes *RFWD2* and *FBXO40* (Glessner *et al*. 2009). A more recent study focused on rare *de novo* copy number variants in ASD families from the Simons Simplex Collection identified a duplication of the DUB gene *USP7* (Sanders *et al*. 2011). The function of these genes in brain development and synaptic plasticity remains to be clarified. However, mutation of the *Uba6* gene encoding an E1 ubiquitin‐activating ligase has recently been shown to result in phenotypes reminiscent of ASD mouse models, including increased dendritic spine density, altered levels of Shank3 and Ube3a, and behavioral deficits including anxiety, reduced social interaction, and impaired communication (Lee *et al*. 2013, 2015). Additional studies should address the question of whether phenotypes related to ASD/ID are common neurobiological consequences of UPS gene mutation.

## Problematic proteostasis in ASD/ID

As large‐scale genetic studies continue to identify novel mutations linked to ASD/ID, it is becoming essential to understand the functional consequences of these mutations. Studies in mutant mouse models suggest that synaptic protein synthesis is dysregulated in several genetic causes of ASD/ID. Considering the clear functional connection between protein synthesis and breakdown, it is not unreasonable to suspect that changes in UPS function would result in similar pathology (Fig. 1a). Evidence from the studies of *UBE3A* mutation illustrate that changes in UPS activity can lead to the multiple pathological changes seen in ASD/ID. However, whether other UPS gene mutations lead to neuropathology reminiscent of ASD or ID remains to be determined.

Another important question is whether disorders that arise because of disruption of protein synthesis also result in changes in UPS function. In this case, pathological changes would not be as a result of changes in protein levels *per se*, but rather an increase in protein turnover (Fig. 1b). This could impair synaptic function by increasing the ratio of new to old proteins, which could have a drastic impact on regulation and function. Alternatively, the compensatory change in the UPS could lead to aberrant breakdown of inappropriate target proteins, or altered ubiquitin‐regulated trafficking of these targets. Teasing this apart would be of particular importance for guiding potential treatment strategies. Indeed, it is possible that the alterations in protein synthesis and compensatory changes in UPS function could contribute to different symptom domains of ASD/ID. Examination of the disruptions that occur in the collective process of proteostasis may therefore be an important next step in understanding the pathogenesis of ASD/ID.
